# Barriers and facilitators to cultural competence in rehabilitation services: a scoping review

**DOI:** 10.1186/s12913-017-2811-1

**Published:** 2018-01-15

**Authors:** Viviane Grandpierre, Victoria Milloy, Lindsey Sikora, Elizabeth Fitzpatrick, Roanne Thomas, Beth Potter

**Affiliations:** 1 Children’s Hospital of Eastern Ontario Research Institute, 401 Smyth Road, Ottawa, ON K1H 8L1 USA; 2 University of Ottawa, Roger Guindon Hall, 455 Smyth Road, Ottawa, ON K1H 8L1 USA

**Keywords:** Cultural competency, Health care services, Rehabilitation services, Scoping review

## Abstract

**Background:**

There is an important need to evaluate whether rehabilitation services effectively address the needs of minority culture populations with North America’s increasingly diverse population. The objective of this paper was therefore to review and assess the state of knowledge of barriers and facilitators to cultural competence in rehabilitation services.

**Method:**

Our scoping review focused on cultural competence in rehabilitation services. Rehabilitation services included in this review were: audiology, speech-language pathology, physiotherapy, and occupational therapy. A search strategy was developed to identify relevant articles published from inception of databases until April 2015. Titles and abstracts were screened by two independent reviewers according to specific eligibility criteria with the use of a liberal-accelerated approach. Full-text articles meeting inclusion criteria were then screened. Key study characteristics were abstracted by the first reviewer, and findings were verified by the second reviewer.

**Results:**

After duplicates were removed, 4303 citations were screened. Included articles suggest that studies on cultural competence occur most frequently in occupational therapy (*n* = 17), followed by speech language pathology (*n* = 11), physiotherapy (*n* = 6), and finally audiology (*n* = 1). Primary barriers in rehabilitation services include language barriers, limited resources, and cultural barriers. Primary facilitators include cultural awareness amongst practitioners, cultural awareness in services, and explanations of health care systems.

**Conclusion:**

To our knowledge, this review is the first to summarize barriers and facilitators to cultural competence in rehabilitation fields. Insufficient studies were found to draw any conclusions with regards to audiological services. Minimal perspectives based on patient/caregiver experiences in all rehabilitation fields underscore a research gap. Future studies should aim to explore both patient/caregiver and practitioner perspectives as such data can help inform culturally competent practices.

## Background

According to the latest Census, 20% of Canadians identify themselves as a minority or foreign born [1]. Minority groups are expected to constitute the majority of the United States population by 2044 [2]. Given North America’s increasingly diverse population, cultural competence in rehabilitation services is a major concern [3–5]. While the need for rehabilitation services has an important impact on all individuals and families, cultural minorities experience additional compounding issues. They encounter language barriers, limited social support systems and cultural barriers, all while often undergoing acculturation [6–8]. Such challenges can affect access to care, leading to issues with treatment compliance and outcome success [6, 9]. Immigrants and refugees face the additional challenge of navigating unfamiliar health care systems [10–12]. Such challenges are critical as communication serves as a pillar for optimal outcomes in successful interventions.

Betancourt, Green, Carrillo, and Ananeh-Firempong’s literature review [13] defines cultural competence from a healthcare context as:

"… understanding the importance of social and cultural influences on patients’ health beliefs and behaviors; considering how these factors interact at multiple levels of the health care delivery system; and, finally, devising interventions that take these issues into account to assure quality health care delivery to diverse patient populations" (p.293).

Despite the increasing attention paid to cultural competence, providing culturally competent services can often be challenging for various reasons. First, culture can influence patients’ values, beliefs, and health-related practices [13, 14]. Second, rehabilitation interventions are typically tailored to meet the needs of the majority populations’ cultural values, which as result do not serve all cultural groups [15–17]. A third challenge is related to assessment bias where incorrect interpretations of patients’ competence occurs [18] and can lead to misdiagnosis amongst minority culture populations [19–21]. Other challenges stem from the influence of culture on patients' responses from the time of diagnosis to treatment. For example, parents may seek to conceal their child’s disability if their culture dictates that disabilities are a source of shame [6, 22]. As a result, parents from some cultural backgrounds may decline an intervention or keep disabilities hidden when in public, thereby limiting quality of life.

An evaluation of whether services effectively address the needs of minority culture populations is therefore required to improve cultural competence in rehabilitation services. Before such an evaluation can take place, there needs to be an understanding of how culture can affect services [23]. Yet, experts have stated that research in cultural competence in the rehabilitation fields is often outdated, anecdotal, and may reflect stereotypical views [20, 24]. Additionally, there appears to be a need for evidence-informed culturally competent services. For example, Aboriginal Early Childhood Development practitioners and parents have expressed frustration about the lack of culturally appropriate assessment tools [19, 21, 25]. Without culturally competent interventions, chances for optimal outcomes may become reduced.

This review was therefore undertaken to review and assess the state of knowledge with respect to barriers and facilitators of cultural competence in rehabilitation services. In order to address this objective, this review considered literature from several fields within the broad area of rehabilitation services. This included services in both adults and pediatric care. The research question addressed in this review was: What are the barriers and facilitators to cultural competence in rehabilitation services?

## Methods

A scoping review methodology was employed. Scoping reviews involve a thorough examination of literature on a specific area of research. As the goal is to provide an overview of evidence as opposed to assessing the evidence, quality appraisals are often omitted [26, 27]. This research was informed by Arksey & O’Malley’s [26] methodological framework for scoping reviews. This methodological framework consists of 5 stages: 1) formulating a research question; 2) identifying appropriate studies with a search strategy by examining electronic databases, and reference lists; 3) selecting eligible studies by creating inclusion and exclusion criteria which can then be applied at the article screening level to determine relevance; 4) recording and categorizing key results (e.g. location of study, intervention, comparator, study populations, study objectives, outcome measures, results, etc.); 5) summarizing and disseminating the results through tables and charts.

In addition, our review was guided by the Preferred Reporting Items for Systematic Reviews and Meta-Analyses (PRISMA) statement [28], a checklist that is intended as a guideline for the reporting of systematic reviews but has broader applicability across other types of knowledge synthesis studies.

### Definitions

The conceptualization of cultural competence, sociocultural barriers, and rehabilitation services was used to guide the study selection criteria. The conceptualization of *cultural competence* varies widely in different fields. For the purposes of this research, it was defined in a healthcare context according to Betancourt et al.’s [13] definition previously provided. As cultural competence is a goal in healthcare services, it is important to understand factors that hinder or facilitate its development, maintenance, and improvement. Betancourt et al. [13] state that a critical component of cultural competence is understanding that social factors (e.g. socioeconomic status and environmental factors such as supports, stressors, and hazards) are intricately woven into cultural factors and thus cannot be separated. *Sociocultural barriers* describe this impermeable link. As a result, it is important to understand the social context when describing cultural competence.

In consultation with a librarian (LS) within the health sciences field, the rehabilitation services chosen for this review were: audiology, speech-language pathology, physio/physical therapy, occupational therapy, and nursing articles related to any of these four fields.

### Selection criteria

Eligible articles were considered if they: 1) discussed health care practitioners in rehabilitation and/or recipients of rehabilitation health care services and where appropriate, their caregivers; and 2) reported on perceived barriers and facilitators to cultural competence in the context of practitioner-patient interactions.

There were no age restrictions for participants, however to prevent response bias, articles were excluded if the study population reported external factors that risked influencing their responses (e.g. war victims, refugees, substance abuse, victims of spousal violence, etc.). Individuals with such sensitive external factors may be influenced to give socially desirable responses when providing self-reports [29]. Non-scientific articles (e.g. magazine articles) were also excluded at the screening level.

Finally, due to time limitations and feasibility, all eligible articles were then rescreened to exclude literature reviews, case studies (*n* = 5 or <), commentaries, editorials, conference papers, and posters.

### Search strategy

A search strategy was developed in consultation with a librarian (LS) to identify relevant articles published from the inception of databases until April 2015. This strategy was applied to the following databases: the Medical Literature Analysis and Retrieval System Online (Medline) database, the Excerpta Medica Database (Embase), the Psychological Information Database (PsycINFO), the Cumulative Index to Nursing & Allied Health Literature (CINAHL) database, the Linguistics and Language Behavior Abstracts (LLBA) database, the Communication, Sciences, and Disorders Dome (ComDisDome) database, the Allied and Complementary Medicine Database (AMED), Occupational Therapy Systematic Evaluation of Evidence (OT Seeker) database, and the Physiotherapy Evidence Database (Pedro).

Major concepts in the search strategy were cultural competence, rehabilitation services, and sociocultural barriers and facilitators. A sample of subject headings and key words used in the search strategy include: cultural competence, cultural sensitivity, minority health, physiotherapy, occupational therapy, audiology, nursing, sociocultural barriers, healthcare disparities, and culturally responsive care. Relevant articles found in the field of nursing were screened to ensure that the fields included rehabilitation.

Two independent reviewers (VG and VM) underwent screening training with 10% of the retrieved articles. The reviewers performed abstract screening independently, after which the reviewers met to assess whether calibration was achieved. Disagreements were discussed with a third party (LS) until consensus was reached. After training was completed, the reviewers applied the eligibility criteria to retrieved titles and abstracts by using a liberal-accelerated approach [30]. This approach consists of two levels of screening. In level one, the first reviewer screened all citations, and a second reviewer screened all excluded citations. In level two, for those titles and abstracts not excluded by both reviewers, full text articles were then screened against the inclusion criteria by both reviewers independently to determine eligibility. Reviewing literature beyond the search strategy involved screening the bibliographies of eligible articles against the inclusion criteria.

A data abstraction form was piloted amongst a random sample of 10% of included articles to see whether the content was sufficient to answer the research questions. Abstracted items included: study characteristics and outcomes related to the barriers and facilitators of cultural competence in rehabilitation services. This pilot was performed by the same independent reviewers (VG and VM). All remaining articles were abstracted using the improved form by the first reviewer. Completed forms were then verified by the second reviewer.

## Analysis

In order to assist with collating, summarizing, and reporting the results as per Arksey & O’Malley’s framework [26], data abstraction files were analyzed in NVivo (version 10.1.2), a qualitative software program. A constant comparative coding method was then used to help present an overview of the results. This process was based on Corbin & Strauss’s [31] open, axial, and selective coding methods. One researcher (VG) performed open coding, which typically consists of studying and assigning labels to each passage. Comparisons of these labels were then made to further refine and conceptualize codes. Selective coding was then performed in order to examine similar concepts and collapse similar codes into major themes.

## Results

**Fig. 1:**
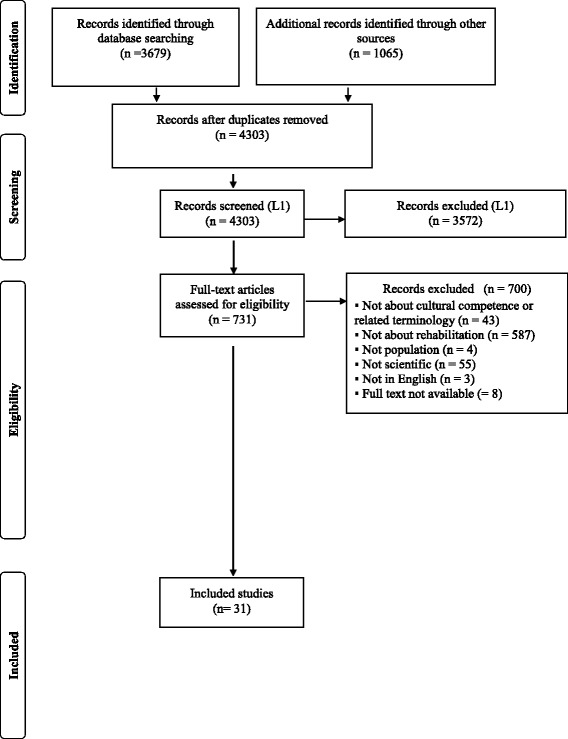
PRISMA Flow Chart

The flow chart in Fig. 1 provides a visual representation of the literature review and search process. After all duplicates were removed, a total of 4303 records were retrieved from the databases as well as additional sources (e.g. recommendations by coauthors, reference lists) were screened at level 1. After excluding 3572 records that did not meet the inclusion criteria, 731 proceeded to a level 2 analysis of the full text. At this level, 700 articles did not meet the criteria for reasons listed in Fig. 1. Of these articles, 8 full text articles could not be retrieved. After all the screenings, only 31 articles were retained. Table 1 describes the eligible articles in detail.

**Table 1:** Study characteristics

Study	Rehabilitation field	Location	Design	No. Of Participants	Study goals
Al Busaidy and Borthwick 2012 [48]	OT	Oman	Interviews	11 Practitioners	Inquired about service provision experiences
Centeno 2009 [33]	SLP	USA	Surveys	33 Practitioners	Inquired about service provision experiences
Dogan et al 2009 [34]^a^	PT	Turkey	Surveys	50 Practitioners	Inquired about service provision and reception experiences
Dressler and Pils 2009 [35]	PT, SLP, OT	Austria	Interviews	28 Practitioners: 1 SLP, 2 OTs, 1 PT; 24 others	Examines practitioners perception of cross-cultural communication experiences
Drolet et al. 2014 [32]	OT, SLP	Canada	Focus groups	43 Practitioners: 21 in health^b^, 22 in social services	Inquired about service provision experiences
Guiberson and Atkins, 2012 [36]	SLP	USA	Survey	154 Practitioners	Inquired about practitioners backgrounds, training, and experiences with service delivery
Jaggi and Bithell 1995 [44]	PT	Bangladesh	Survey	68 Practitioners	Inquired about practitioners experiences, knowledge, and attitudes regarding service delivery
Khamisha 1997 Part 1 & 2 [37, 53]	OT	Glasgow	Survey	94 Practitioners	Inquired about practitioners perceptions, experiences, knowledge, and attitudes regarding service delivery
Kinebanian and Stomph 1992 [46]	OT	Netherlands	Interview	25 Practitioners	Inquired about service provision experiences
Kirkham et al. 2009 [57]	Aud	USA	Survey	103 Practitioners	Inquired about perceptions of speech and language outcome disparities and recommendations to reduce disparities
Kirsh, Trentham and Cole 2006 [61]	OT	Canada	Interviews	14 Consumers	Inquired about minorities’ experiences with receiving services
Kohnert et al. 2003 [38]	SLP	USA	Survey	104 Practitioners	Inquired about service provision experiences
Kramer-Roy 2012 [62]	OT	United Kingdom	Interviews	6 caregivers	Inquired about the service needs of Pakistani families with disabled children
Kummerer and Lopez-Reyna 2006 [60]	SLP	USA	Interviews	14 caregivers	Explored the views and beliefs of language development, disabilities, therapy experiences of Mexican immigrant mothers
Lee, Sullivan and Lansbury 2006 [58]	PT	Australia	Interviews & Observations	6 Practitioners	Explored practitioners strategies with service delivery
Lindsay et al. 2012 [12]	OT, PT	Canada	Interviews & Focus Groups	13 Practitioners & coordinators: 2 PTs, 2 OTs, 9 others	Inquired about service provision experiences
Lindsay et al. 2014 [39]	OT	Canada	Interviews	17 Practitioners	Explored practitioners strategies with service delivery
Maul, 2010 [54]	SLP	USA	Interviews	9 Practitioners	Explored cultural competency skills in practitioners
Munoz 2007 [55]	OT	USA	Interviews	12 Practitioners	Explored practitioners’ perceptions of culturally competent service delivery
Nelson and Allison 2007 [43]	OT	Australia	Part 1: Interviews & focus groups Part 2: Surveys	Part 1: 25 Stakeholders including 8 caregivers Part 2: 50 Practitioners	Explored practitioners’ perceptions of culturally competent service delivery
Nelson, Allison, and Copley 2007 [49]	OT	Australia	Part 1: Survey Part 2: Focus groups & Interviews	Part 1: 50 Practitioners Part 2: 25 Stakeholders including 8 caregivers	Inquired about service provision and reception experiences
Nelson et al. 2011 [56]	OT	Australia	Survey & Workshop discussion	41 Practitioners	Inquired about service provision experiences
Phipps 1995 [40]	OT	Australia	Survey	65 Practitioners	Inquired about service provision experiences
Phoon and Maclagan 2009 [50]	SLP	Malaysia	Survey	38 Practitioners	Explored practitioners experiences with using assessments
Roseberry-McKibbon and Eicholtz 1994 [41]	SLP	USA	Survey	1145 Practitioners	Inquired about service provision experiences
Roseberry-Mckibbon, Brice and O’Hanlon, 2005 [42]	SLP	USA	Survey	1736 Practitioners	Inquired about service provision experiences
Stedman and Thomas 2011 [51]	OT	Australia	Interviews	7 Practitioners	Inquired about service provision experiences
Watts and Carlson, 2002 [52]	OT	Australia	Interviews	8 Practitioners	Inquired about practitioners’ experiences, perspectives and recommendations regarding service provision
Williams and McLeod 2012 [45]	SLP	Australia	Survey	128 Practitioners	Inquired about practitioners’ experiences and perspectives regarding service provision
Yang et al. 2006 [47]	OT	Singapore	Interviews	9 Practitioners	Explored the applicability of OT frameworks in Oman context
Yeowell 2010 [59]	PT	England	Interviews	6 Patients	Inquired about the service needs of Pakistani women

*OT* Occupational Therapy, *PT* Physiotherapy, *SLP* Speech-language pathology, *Aud* Audiology

^a^This study did not have/require ethical clearance

^b^This study does not specify the number of practitioners per rehabilitation field

### Study characteristics

Of the 31 eligible articles, 17 were in occupational therapy (OT), 11 in speech-language pathology (SLP), six in physiotherapy (PT), and one in audiology (Aud). Four of these articles reported on multiple rehabilitation fields (note: 3 studies reported on multiple rehabilitation service). Table 2 displays the number of participants within each field. Fifteen articles discussed experiences within a pediatric-context: one in audiology, one in physiotherapy, six in occupational therapy, and eight in speech-language pathology (note: one article had OT and SLP participants). Seventeen articles used qualitative methods, 12 used quantitative, and two used mixed methods. The majority of these studies took place in Canada and the USA, with other study locations in Malaysia, Austria, Germany, Australia, England, Netherlands, Scotland, Bangladesh, Oman, Singapore, and the United Kingdom (Table 1).

**Table 2:** Number of participants per rehabilitation field

Field	# of health care practitioners	# of patients/caregivers
Occupational therapy	343	28
Audiology	103	N/A
Speech language pathology	3348	14
Physiotherapy	127	6

*N/A* not applicable due to no study availability on patient/caregiver perspectives & Drolet [32] was excluded from the count as it did not specify the number of practitioners in each field

### Practitioner perspectives

We identified a multitude of barriers and facilitators to service delivery and reception, which is reported below from the perspectives of practitioners and patients/caregivers. Table 3 displays and compares various common themes reported by the practitioners and patients/caregivers of the reviewed articles. Though overlap occurs between categories, the results provide an overview in understanding how service delivery and reception can be impacted by diversity.

**Table 3:** A comparison of barriers and facilitators between patients/caregivers’ and practitioners perspectives in rehabilitation services

	OT HCP	OT PTs	PT HCP	PT PTs	SLP HCP	SLP PTs	Aud HCP	AUD PTs
Barriers								
Language barriers	✔	✔	✔	✔	✔	✖	✖	✖
Limited resources	✔	✖	✔	✔	✔	✖	✖	✖
Influence of cultural difference	✔	✖	✔	✖	✔	✖	✖	✖
Facilitators								
Cultural awareness amongst practitioners	✔	✔	✖	✔	✔	✔	✖	✖
Cultural awareness in services	✔	✔	✔	✔	✔	✖	✔	✖
Explanations of health care systems	✔	✔	✔	✔	✖	✔	✖	✖

*OT* Occupational Therapy, *PT* Physiotherapy, *SLP* Speech-language pathology, *Aud* Audiology, *HCP* Health care practitioners, *PTs* Patients, ✔ Confirmed in studies, ✖ No study availability

### Barriers reported by practitioners

Practitioners described many barriers in providing rehabilitation services to minority culture service patients. Three major categories emerged from the data: The effect of language barriers, the influence of cultural differences on service delivery, and limited resources to facilitate culturally competent care. Table 4 provides an overview of the primary barriers experienced by both practitioners and patients/caregivers and how they influenced various aspects of healthcare delivery.

**Table 4:** Overview of the primary barriers and how they influenced various aspects of healthcare delivery/reception

Primary barriers to culturally competent care	Areas of health care service delivery/reception affected
Language barriers	• Practitioner-patient/caregiver communication • Establishment of rapport • Information provision and instruction • Engagement in intervention/therapy
Cultural barriers	• Practitioner-patient/caregiver communication • Establishment of rapport • Diagnosis • Decision-making on treatment • Engagement in intervention/therapy
Limited resources	• Practitioner-patient/caregiver communication • Establishment of rapport • Diagnosis • Assessments • Engagement in intervention/therapy

#### The effect of language barriers

Language barriers were reported by speech language pathologists, physical therapists, and occupational therapists. Practitioners unable to speak the language of their patients felt language barriers limited their abilities to provide information and instructions [12, 32–42]. Not being able to communicate effectively with service recipients was also said to impact the development of effective relationships [43] and as a result, it took longer to establish rapport [39]. Difficulties in service delivery were reported to also arise when a child’s primary caregiver (typically who is most knowledgeable of the child’s behaviors) was unable to speak the language, leaving the other parent to act as the family spokesperson [12]. Finally, language barriers were also said to hinder and sometimes impede therapy delivery [35] and potentially affect treatment compliance [44].

#### The influence of cultural differences on service delivery

Speech language pathologists, physical therapists, and occupational therapists reported cultural differences affected service delivery. In a pediatric context, cultural differences were seen in child-rearing strategies. Interacting with fathers was reported to be challenging due to gender attitudes varying across cultures [45]. Occupational therapists also identified cultural differences in play. Therapists spoke of cultures where parents do not play with their children. This was seen to complicate service delivery as therapists felt conflicted about encouraging parents to use play in therapy [39].

Cultural differences were also said to occur in the caregiver’s views of disability, independence, decision-making, and gender roles. Differing views of disability sometimes affected treatment compliance. For example, an occupational therapist participating in a focus group stated:

“Some recommendations you’ll give a child for safety concerns or you provide a child with equipment so they’re better supported so feeding could be more successful and more in a safe way and yet they still have a lot of [difficulty] culturally [with] their food, they want to be feeding that even though a different food is suggested”. Lindsay et al. [12], pp. 2011.

Views of independence were also said to vary across cultures [12, 39, 40, 46, 47]. Western-based practices value the promotion of independence however the assumption that this is a universal value has limited the provision of culturally competent care. Yang [47] described challenges experienced by occupational therapists where patients did not believe achieving independence was important as it was the responsibility of their families or maids to care for their children. Additionally, activities of daily life used in occupational therapy were not seen as meaningful within some cultures [40, 47].

Differences in decision-making were documented in several studies [12, 39, 45, 47]. In particular, patients were seen to be reluctant in making decisions, as they believed such decisions should be left to experts.

Finally, cultural differences in gender roles were seen to impact service delivery [12, 35, 39, 48]. In some cases, male service recipients requested male practitioners [35, 48]. In a pediatric case, therapists experienced challenges in requesting information regarding the needs and abilities of children, as mothers (typically the primary caregiver) stayed silent during assessments since fathers were the family spokesperson.

#### Limited resources to facilitate culturally competent care

Speech language pathologists, physical therapists, and occupational therapists cited limited resources in providing culturally competent care. This included Western-based practices, linguistically-relative materials, lack of bilingual practitioners, lack of interpreters, and a lack of sufficient training and/or education.

Several studies described how Western-based notions of rehabilitation complicated service delivery [39, 46, 48, 49]. For example, service models adhering to Western values typically promote independence, which as previously shown, was not always considered to be important by some cultural groups. Barriers also included culturally and/or linguistically-relative materials, assessments, and treatments. The lack of these resources was frequently cited as a barrier to culturally competent service delivery [12, 33, 35, 36, 38–42, 46, 47, 49–52].

In terms of linguistically-relative materials, offering information and recommendations to service recipients in English created challenges in providing therapy [35]. These limitations affected relationship-building opportunities [12]. Regarding service materials, several studies discussed challenges with providing appropriate assessment materials, treatment planning, treatment materials, and treatment goals [33, 36, 38–40, 42, 46, 47, 49, 50, 52]. In particular, studies reported a lack of appropriate assessment/screening instruments creating barriers to culturally competent service delivery [33, 36, 38, 40–42, 49, 50, 52]. Such limitations become increasingly worrisome when there are already difficulties in differentiating a language difference from a language disorder [41, 42].

Difficulties in the provision of culturally competent services were also attributed to a lack of bilingual practitioners or practitioners who speak their clients’ language [33, 36, 38, 41, 42], lack of available interpreters [41], and practitioners receiving minimal or no training and/or education on servicing minorities [12, 33, 38–41, 45, 53].

### Facilitators reported by practitioners

Practitioners described a variety of facilitators in providing rehabilitation services to minority culture patients. Three major categories emerged from the data: Increasing cultural awareness, fostering a culturally competent work environment, and explaining healthcare to minority culture patients. Table 5 provides an overview of the primary facilitators experienced by both practitioners and patients/caregivers and how they influenced various aspects of healthcare delivery.

**Table 5:** Overview of the primary facilitators and how they influenced various aspects of healthcare delivery/reception

Primary facilitators to culturally competent care	Impact on health care service delivery/reception
Cultural awareness amongst practitioners	• Helped establish rapport • Helped with provision of appropriate care/therapy • Helped to tailor care/therapy when needed • Helped with understanding patient/caregiver health-related goals
Cultural awareness in services	• Improved practitioner-patient/caregiver communication • Helped establish rapport • Increased attendance and compliance • Helped to learn about patients’/caregivers’ values and needs • Helped diminish negative experiences • Created a comfortable atmosphere • Helped support patients/caregivers with long-term treatment management
Explanations of health care systems	• Increased patient/caregiver understanding of available services and resources • Increased patient/caregiver understanding of available funding • Increased patient/caregiver understanding of available support networks • Increased patient/caregiver understanding of benefits of treatment and compliance

#### Increasing cultural awareness

This category emerged from data discussing methods that enabled culturally competent care. Asking questions was one method that helped determine cultural differences which might require tailoring care. Inquiring about patients’ day-to-day practices was seen as a helpful strategy for learning about cultural differences and providing appropriate therapy [39, 46, 51, 54]. Asking about family roles may help with service provision. For example, according to Nelson’s [49] study, therapists experienced difficulties in communicating with the same caregiver as Indigenous patients often have multiple caregivers or extended families. This led to uncertainty about compliance as it was difficult to know if the information was being understood and transferred at home.

Understanding patients’ cultural backgrounds was viewed as important in many studies [39, 49, 51, 54]. Such knowledge helped practitioners better understand patient goals and offer more appropriate recommendations [39]. Learning about the histories of cultural groups was also seen as a facilitator to providing culturally competent care. For example, discrimination and marginalization experienced by Indigenous Australians may lead to patients feeling disempowered and wary of government services and may effect attendance [49, 52]. Strategies used by practitioners to address the impact of such histories include environmental considerations, such as conducting therapy sessions outdoors or in areas where patients are more comfortable [52].

Learning about the role of religion and traditional healing methods was also seen as an important facilitator. Unlike Western medicine where illness and religion are separate entities, cultures exist where religious and traditional healing roles govern perceptions of illness as well as every day practices [46, 48]. Having an awareness of the ties between religion and health may allow practitioners to better tailor care to meet the needs of their minority patients. Practitioners seeking to gain knowledge about cultural differences, cultural histories, and/or the roles of religion and traditional healing methods can educate themselves with the use of books and media [33, 37, 40].

Establishing meaningful relationships, engaging in cross-cultural encounters, having respect for cultures, and being reflective were also identified as approaches to developing cultural awareness. Establishing a meaningful relationship was seen as an essential factor for ensuring the provision of appropriate and successful interventions [43, 51]. Such relationships can result in patients providing relevant information needed to develop appropriate treatment plans. This involves knowing how to formulate questions, although this was seen as challenging as patients sometimes limit their responses to ‘yes’ or ‘no’ [51]. Approaches to establishing and maintaining relationships include inquiring about patients’ cultural backgrounds, learning certain key words and phrases in the patients’ primary language, understanding the patient’s values, and being mindful of verbal and non-verbal communication [39, 40, 43, 52, 54, 55]. Having respect for cultures can also facilitate beneficial exchanges with patients [43].

Engaging in cross-cultural encounters was also viewed as a useful strategy to developing cultural awareness. This can involve creating links with cultural agencies, attending cultural events, interacting with communities, or simply engaging in day-to-day interactions with culturally diverse individuals [49, 54–56].

Finally, being reflective was noted by numerous studies as an important requirement for developing cultural awareness. This involved practitioners examining their own cultural identity, values, prejudices, biases, and/or assumptions and the influence it can have on service delivery [39, 49, 51, 55, 56].

#### Fostering a culturally competent work environment

Numerous studies called for a need to foster culturally competent work environments. One approach for achieving this goal was to have a more diverse workforce [57]. Flexibility was also seen as an important trait in providing services to minority culture patients [12, 39, 43, 49]. Flexibility helped create a better understanding of patients’ day-to-day activities [39] and build relationships [12]. One strategy to becoming flexible can involve increasing appointment time when working with minority culture patients [12].

Another approach to fostering a culturally competent work environment was training and/or education on providing services to minorities [33, 34, 42, 44, 52, 57]. Such training/education was often cited as a need [49, 53, 56].

Having registration forms that collect linguistic and cultural background was seen as a helpful method to providing appropriate care [12]. Using the services of other professionals, such as colleagues with experience in working with cultural minorities, interpreters, and cultural liaisons was sometimes seen as helpful [12, 33, 38, 40, 41, 43–45, 49, 52, 56, 57]. Working with interpreters however was also reported as challenging in terms of cost, increased time and effort with interactions, trust issues, minimal knowledge of professional jargon, and creating barriers with building rapport [12, 54, 58]. Using colleagues as interpreters was also flagged as inadvisable due to the lack of training, which certified interpreters are required to undergo [58].

Rehabilitation services that incorporate family members into practice was seen as a useful strategy to help build culturally competent services as there are cultures where immediate and extended family members can have a significant role in a patient’s life [40, 52]. Another strategy that recognizes the importance of relationships was using small group sessions in therapy. For example, Australian Indigenous children may experience a sense of shame for having to see a therapist and having small group sessions can help diminish such negative experiences [43]. Services that network with cultural agencies and/or organizations was reported as another useful strategy that helped with initial patient encounters, developing relationships, and attaining consistent follow-up [40, 43, 51].

Specific strategies to facilitating culturally-competent work environments were also reported. Matching practitioners with patients of similar cultural background was one recommendation [12, 40]. Another approach involved the use of culturally sensitive materials [35, 52, 56]. For example, use of pictorial images to help improve communication was reported to help patients who do not speak the service language [56].

Specific strategies for assessments and treatments were also reported. Tailoring assessments and treatments can first involve gathering cultural data through interviews and observations [48, 54, 55]. Gathering such information can be challenging, however there were a variety of solutions identified for overcoming this barrier: using pauses (e.g. giving time for patients to respond), soft voices, informal language, and/or non-verbal media such as pictorial brochures to support communication [52, 56]. Next, modifications to care can occur with the use of: interpreters, tests developed for multicultural populations, informal assessments (e.g. language samples, checklists), translated materials, toys familiar to children, communication equipment (e.g. video conferencing materials) for rural and remote patients, and selecting culturally-meaningful treatments [40, 45, 46, 50–52, 54, 55, 58, 59].

Finally, practitioners called for more research on cultural differences. Such information would help inform culturally competent practices [52, 56].

#### Explaining healthcare to minority culture patients

Supporting minority culture patients navigating the health care system was identified as an important feature for providing culturally competent care, as many may not know about the resources available to them. Helping patients understand the health care system can include providing home visits, connecting them to resources, explaining how equipment is funded, and/or offering personalized support networks [12, 39]. Explaining perceptions of disability in the country where the service is being provided was also highlighted as important to helping patients understand the health system as there are cultures where disability is stigmatized and hidden [39].

Finally, explaining what is involved in assessments and treatments was also felt to be important by practitioners. This can be achieved by using appropriate terminology, written material with simple language, cultural liaisons, and/or information sessions [39, 49, 60].

### Patient/caregiver perspectives

Although results regarding patient/caregiver perspectives on culturally competent care were limited as only five studies enrolled patient/caregiver participants, a variety of barriers and facilitators were nonetheless found.

### Barriers reported by patients/caregivers

Patients/caregivers described a variety of barriers to receiving high quality rehabilitation services. Two major themes emerged from the data: The effects of language and cultural barriers and the effects of limited resources in services.

#### The effects of language and cultural barriers

Patients described instances of being unable to communicate thoughts and feelings [59]. There were also descriptions of service recipients experiencing attitudinal issues whereby practitioners used unfamiliar language. This resulted in service recipients questioning whether they were experiencing discrimination due to their minority status [61]. Language barriers also affected caregivers understanding of meaningful treatment goals that would help improve development outcomes [60].

#### The effects of limited resources in services

Services that did not provide interpreters and assumed that the patient will bring someone who can translate was seen as a barrier. For example, not having an interpreter was noted to have affected attendance in one study [59].

Another barrier was the use of written information during service provision. Even if materials were translated, some service recipients noted that they could not read in their native language [59].

### Facilitators reported by patients/caregivers

Patients/caregivers described a variety of facilitators in receiving culturally competent rehabilitation services. Three major themes emerged from the data: cultural awareness amongst practitioners, cultural awareness in services, and explanations of health care systems.

#### Cultural awareness amongst practitioners

According to patients/caregivers, a key facilitator to receiving culturally competent services was having practitioners who posessed cultural awareness. This involved practitioners developing an understanding of culture, including cultural history, how it affects patients/caregivers’ everyday practices (e.g. ritual occupations and traditions) and making an effort to be non-judgemental [43, 49, 61, 62]. Suggestions for gaining such knowledge were to spend time with different cultural groups and have conversations with professionals with cultural experience or cultural liaisons [49].

Cultural awareness also involves recognizing there are cultural differences in the perceptions of disability, such as etiology of the disability [61, 62]. Differences also occur in activities such as play. Discussing service recipients’ views of play may help improve the success of interventions as therapy can be better tailored to reflect the caregivers’ everyday environment [62].

Patients/caregivers also spoke of the importance of relationships with practitioners and the need to work in partnership within that relationship [43]. They reported how important it was to have practitioners share information about their lives (e.g. social, cultural, historical aspects) [61]. Patients/caregivers also described the need to have the same therapist in order to facilitate long-term relationships [43]. Having a practitioner with the same cultural background and/or sex can help establish a relationship as the practitioner may be seen as someone who would be familiar with taboos. However it should be noted that some patients also expressed concerns regarding this facilitator in terms of maintaining confidentiality within their communities [61].

Exploring caregivers’ expectations of development was also valuable as knowledge of such interpretations can help facilitate effective therapy strategies. Without such information, compliance may be affected as service recipients may not understand the value of treatment plans [62]. Eliciting information on expectations of language milestones can include encouraging story-sharing with the use of videotapes and/or journal entries [60]. Although the strategies mentioned in this section can help develop and improve relationships, caregivers reported that the personal qualities of practitioners were also essential to developing cultural awareness [49].

#### Cultural awareness in services

Patients/caregivers expressed an appreciation for services that incorporated cultural awareness into practice protocols. This involved services that used culturally appropriate materials and tailored care to meet the needs of minority patients/caregivers.

Culturally appropriate assessment and intervention materials were valued by service recipients as such resources were typically developed for North Americans [49, 61]. To overcome this limitation, one suggestion was to use observations to complement assessments. Another suggestion was the use of photographic or visual home programmes for those who do not have strong literacy skills [49]. An alternative is the provision of written instructions with pictures [59].

Tailoring care involved understanding patient needs. Patients indicated a preference for having practitioners of the same gender and for single-sex group sessions. Tailoring care in this manner may have a positive effect of compliance and attendance [59]. Having longer appointment times for patients who do not speak the service language was also recommended to facilitate culturally competent service provision [59]. One patient discussed how speaking English as a second language takes time and that it would be helpful for practitioners to be aware of this. To ensure comprehension, this patient recommended practitioners to go slowly:

“If I’m talking English and you’re speaking English, I’ve got to take it in as English, but if I don’t speak good English, when you’re speaking in English I’ve got to take it in and translate it in my head and translate it into your language and then back into English to speak it. Yes. So I think you need to give them space and check they’ve understood before they go on to the next sentence. That would help.” Yeowell [59], pp. 261.

Caregivers may also benefit from services such as support groups that include participants cultural/religious backgrounds. This strategy can help support caregivers with long-term treatment management [62].

#### Explanations of health care systems

Patients/caregivers expressed the need for understanding rehabilitation services. Specifically, the purpose of therapy, how long it will take, the roles of family members in supporting it, and the benefits of compliance, particularly if aspects of treatment (e.g. exercise) are not a part of their culture [49, 59, 60]. Practitioners who possess cultural awareness and are able to offer such explanations are therefore in a better position to provide culturally competent care.

## Discussion

### Summary

Increasing diversity has called attention to the need for culturally competent health care services. This scoping review sought to identify practitioner’ and patient’/caregivers’ perspectives on barriers and facilitators to cultural competence in rehabilitation services. Three major barriers emerged from the data reporting on practitioner perspectives: The effect of language barriers, the influence of cultural differences on service delivery, and limited resources to facilitate culturally competent care. Major facilitators identified were: increasing cultural awareness, fostering a culturally competent work environment, and explaining healthcare to minority culture patients. Two major barriers emerged from data on patient’/caregivers’ perspectives: the effects of language and cultural barriers and the effects of limited resources in services. Major facilitators were: cultural awareness amongst practitioners, cultural awareness in services, and explanations of health care systems.

### Comparing barriers and facilitators in pediatric services with adult services

There was much overlap in the barriers and facilitators reported by both adult and pediatric services, however there were a few notable differences. Barriers listed in articles discussing pediatric care were reportedly due to the influence of cultural differences. Specifically, cultural differences in child rearing [45] and play [39] presented challenges to intervention practices. Differences in the understanding of disability were also seen to impact service delivery. Practitioners reported how perceptions of disabilities were difficult to manage as these views sometimes extended to expectations of how it can be fixed as opposed to managed [12, 45].

Differences in facilitators for pediatric services included cultural awareness in services. Specifically, the call for hospitals to collect information on cultural backgrounds upon registration [12] was unique to a pediatric study and was not seen in adult services. In addition, explanations of health care systems was identified as a facilitator unique to pediatric services [12, 39, 49]. Knowledge of these barriers and facilitators may help rehabilitation practitioners better tailor care when working with multicultural families of children with disabilities.

### Comparing patient’/caregivers’ perspectives with practitioner perspectives

Five studies investigated patient’/caregivers’ perspectives regarding service needs and experiences (note: the Nelson articles [43, 49] stemmed from one study and were therefore counted once here). Sample sizes in these studies were smaller in comparison to practitioner participants. This highlights a need for more research on minority patient’/caregivers’ perspectives. Research exploring dual perspectives of both practitioners and patients/caregivers could be compared, thereby providing a rich source of information which could be used to inform practice guidelines.

The majority of studies on practitioners investigated their perspectives and experiences with service delivery to multicultural populations. Two studies focused specifically on therapy outcome disparities and applicability of a Western therapy framework in a foreign country [47, 57]. Interestingly, there were more remarks about barriers than facilitators in patient’/caregivers’ perspectives compared to practitioner perspectives. This finding suggests a need to investigate feasible solutions to known barriers when working with a diverse population.

A comparison of barriers and facilitators revealed similarities between patient’/caregivers’ and practitioners perspectives. Both practitioners and patients/caregivers experienced service limitations stemming from language barriers and a lack of resources. Facilitators suggested by both practitioners and patients/caregivers included having practitioners who possess cultural awareness and offer explanations of health care systems, as well as having services that incorporate cultural awareness into practice protocols.

### Comparing results across disciplines

In examining results across disciplines, there appeared to be strong consensus regarding barriers as reported by practitioners. All rehabilitation fields with the exception of audiology described barriers according to the three themes presented here. The study in audiology however mainly investigated disparities in speech and language therapy outcomes and as such, it is difficult to know what the state of barriers are in this field.

Differences across disciplines were more noticeable in facilitators as reported by practitioners. The theme of increasing cultural awareness was discussed extensively in occupational therapy studies. It also emerged in one speech language pathology study, although it did not appear in the remaining rehabilitation disciplines with the exception of one physiotherapy study that reported on patient’/caregivers’ perspectives. Only studies in occupational and physiotherapy described a need for explanations of healthcare systems. This theme was however discussed in a speech-language pathology study on patient’/caregivers’ perspectives. The need for cultural awareness was discussed in every discipline with the exception of patient’/caregivers’ perspectives in audiology.

### Limitations

Our review was not without limitations. First, the lack of research in audiology resulted in exploring disciplines beyond the original focus of this paper. Second, the search strategy was restricted to English articles. As such, perspectives are not globally representative. Third, screening articles beyond the search strategy was limited to scanning bibliographies of eligible studies due to time constraints. As a result, there is a possibility that articles were missed. Fourth, the review excludes the perspectives of vulnerable groups (e.g. war victims, refugees). Nonetheless, considerations for how to engage in culturally competent rehabilitation services were provided, along with suggestions for how to overcome common barriers when interacting with multicultural populations.

## Conclusion

This scoping review summarized barriers and facilitators to cultural competence in rehabilitation services. While several studies on this topic were found in the fields of speech-language pathology, physiotherapy, and occupational therapy, insufficient studies were found to draw any conclusions with regards to audiological services. Minimal perspectives based on patient/caregiver experiences in this field underscore a research gap. Future studies should aim to explore both patient/caregiver and practitioner perspectives on service provision and reception as such data can help inform evidence-based practices when providing services to cultural minorities.

## Abbreviations

Aud: Audiology
HCP: Health care practitioners
OT: Occupational Therapy
PRISMA: Preferred Reporting Items for Systematic Reviews and Meta-Analyses
PT: Physiotherapy
PTs: Patients
SLP: Speech-language pathology
